# Characteristics and outcomes of acute hepatitis of unknown etiology in Egypt: first report of adult adenovirus-associated hepatitis

**DOI:** 10.1007/s15010-022-01945-1

**Published:** 2022-11-05

**Authors:** Haidi Karam-Allah Ramadan, Ibrahim M. Sayed, Amal A. Elkhawaga, El-Zahraa M. Meghezel, Ashraf A. Askar, Abdelmajeed M. Moussa, Asmaa O. B. S. Osman, Azza Abo Elfadl, Walaa A. Khalifa, Ahmed M. Ashmawy, Mohamed A. El-Mokhtar

**Affiliations:** 1 Department of Tropical Medicine and Gastroenterology, Faculty of Medicine, Assiut University, Assiut, Egypt; 2 Department of Medical Microbiology and Immunology, Faculty of Medicine, Assiut University, Assiut, Egypt; 3 Present Address: Department of Biomedical and Nutritional Sciences, University of Massachusetts Lowell, Lowell, MA United States; 4 Department of Tropical Medicine and Gastroenterology, Faculty of Medicine, Sohag University, Sohag, Egypt; 5 Department of Internal Medicine, Faculty of Medicine, Sohag University, Sohag, Egypt; 6 Department of Tropical Medicine and Gastroenterology, Faculty of Medicine, Aswan University, Aswan, Egypt; 7 Clinical Pathology Department, Faculty of Medicine, Assiut University, Assiut, Egypt; 8 Department of Internal Medicine, Faculty of Medicine, Assiut University, Assiut, Egypt

**Keywords:** Acute hepatitis, AHUE, Adenovirus, Non-A–E hepatitis, Acute liver failure, Adult, Egypt

## Abstract

**Purpose:**

Several outbreaks of acute hepatitis of unknown etiology (AHUE) in children were reported in 2022 in many countries, with adenovirus identified as the etiological agent in most of them. We aimed to evaluate the characteristics and outcomes of AHUE cases in Egypt.

**Methodology:**

Hospitalized patients with acute hepatitis were included in the study. Drug-induced, alcoholic hepatitis, autoimmune hepatitis, and Wilson’s disease were identified either by medical history or by routine laboratory diagnosis. Molecular and serological approaches were used to investigate common viral causes of hepatitis, such as hepatitis A–E viruses, cytomegalovirus, Epstein–Barr virus, herpes simplex viruses (HSV1/2), adenovirus, parvovirus B19, and coxsackie virus.

**Results:**

A total of 42 patients were recruited and divided into two groups: 24 cases of unknown hepatitis after excluding the common causes and 18 cases of known hepatitis. About two-thirds of the patients were male (61.9%), and the mean age was 34.55 ± 16.27 years. Jaundice, dark urine, abdominal pain and diarrhea were recorded at a higher incidence in group 1, while jaundice and fever were frequent in group 2. Fulminant hepatitis occurred in 28.6% of the cases, but the two groups did not differ significantly in terms of patient outcome, duration of hospitalization, ascites, and development of fulminant hepatitis. Adenovirus was detected in five cases (20.8%) in group 1, and one case co-infecting with hepatitis E virus in group 2. Herpes simplex virus 1/2, coxsackie virus, and parvovirus B19 were not detected in any case, while etiologies of 75% of the cases were still not confirmed. One out of the six adenovirus-infected patients died. The outcome significantly correlated with the severity of the liver disease.

**Conclusion:**

This is the first report describing etiologies and characteristics of AHUE cases in Egypt, and interestingly, adenovirus was detected in adults. Further studies are required to determine the prevalence of this newly emerging viral hepatitis pathogens.

## Introduction

Viral hepatitis and paracetamol-induced liver injury are the leading drivers of acute hepatitis and acute liver failure (ALF) worldwide [1, 2]. The etiology of about 20% of ALF cases is cryptogenic [3]. The incidence of indeterminate ALF varies geographically and ranges between 5.5% [4–6] and 50% [7]. Chiefly, hepatitis A–E viruses are responsible for ALF, while herpes simplex virus (HSV), cytomegalovirus (CMV), Epstein–Barr virus (EBV), parvovirus B19, and coxsackie virus can also cause acute hepatitis, rarely [3].

The outcome of ALF and the risk factors for morbidity or mortality may vary according to the etiology. Although hepatitis caused by the A and E viruses usually recovers spontaneously, recent studies from Egypt showed that the hepatitis E virus can cause fatal fulminant hepatitis, especially in elderly and leukemic patients [8, 9]. In contrast, non-A–E virus-induced or drug-induced ALF is associated with high mortality in the short term [3, 10, 11]. Therefore, ALF cases that require liver transplantation must be selected carefully.

In April 2022, 84 cases of acute hepatitis of unknown etiology (AHUE) in children were reported to the World Health Organization: 10 from Scotland and 74 from the United Kingdom. Laboratory diagnosis confirmed that adenovirus and severe acute respiratory syndrome coronavirus type 2 (SARS-CoV-2) were the common etiological agents [12]. By 8 July 2022, 1010 probable cases and 22 deaths were reported across 35 countries [13]. Several reports showed that adenovirus was isolated in most of these children (*n = *209); however, its role in the pathogenesis of this disease was not clarified [14, 15].

In Egypt, studies on the prevalence, characteristics, and outcomes of AHUE are lacking. The aim of this study was to assess the different etiologies, clinical and laboratory characteristics, and outcomes of AHUE patients admitted to Assiut University Hospitals. Surprisingly, we detected adenovirus in adult patients with AHUE. To the best of our knowledge, this is the first report on the association between AHUE and adenovirus in Egypt.

## Materials and methods

### Patients and methods

This study involved patients with acute hepatitis, fulminant hepatitis, or acute-on-chronic liver failure, visiting the Department of Tropical Medicine and Gastroenterology, Al-Rajhi Liver University Hospital, Assiut University, and Sohag University Hospital, from October 2020 to June 2022. The minimal criteria for inclusion in the study were all hospital-admitted patients who fulfilled the criteria of symptomatic acute hepatitis either classical icteric or anicteric in the specified period. Patients with known causes of chronic liver diseases, hepatocellular carcinoma, or other malignancies were excluded. Detailed histories and examinations were obtained. Records of the duration of symptoms, hospitalization, and the outcomes were collected. Liver function tests (LFTs), prothrombin time and international normalized ratio (INR), serum creatinine, complete blood count (CBC), and abdominal ultrasound were performed for all participants. None of the patients showed any clinical symptoms or tested positive for coronavirus disease 2019 (COVID-19).

#### Screening of viral hepatitis

Acute hepatitis A virus (HAV) and hepatitis E virus (HEV) infections were screened by enzyme-linked immunosorbent assay (ELISA) using the anti-HAV immunoglobulin M (IgM) kit (CTK Biotech, USA) and the abia HEV IgM kit (AB Diagnostic Systems GmbH, Berlin, Germany), respectively. Hepatitis B virus (HBV) infections were identified by detecting hepatitis B surface antigen and anti-HBV core IgM using commercially purchased ELISA kits (Prechek Bio Inc., Taiwan). Samples that tested positive for HBV were also screened for anti-hepatitis D virus antibodies using the commercial ELISA kit ETI-ABDELTAK-2 (DiaSorin S.p.A., Italy). Samples were also initially tested for hepatitis C virus (HCV) by detecting anti-HCV antibodies using the fourth-generation HCV TRI-DOT test control (Atlas Link, USA). In addition, HBV and HCV were screened for by quantitative real-time PCR by detecting viral nucleic acids using commercially available PCR kits. Briefly, viral nucleic acids were extracted from serum samples using the QIAamp MinElute Virus nucleic acid extraction kit (Qiagen, Germany), followed by analysis using the artus HBV RG PCR kit (Qiagen, Germany) and the artus HCV RG RT-PCR kit for detecting HBV and HCV, respectively.

Other causes of viral hepatitis were screened using commercial ELISA kits to detect anti-EBV antibodies, anti-CMV IgM, anti-HSV1/2 IgM, anti-parvovirus B19 IgM/IgG, and anti-coxsackie B virus IgM and IgG (Serion ELISA classic, Germany). CMV DNA was tested by PCR, as described previously. The assay can detect as low as 2000 genome equivalents/sample [16]. For detection and quantification of human adenovirus, DNA was extracted from serum samples using the Qiagen Blood kit (Qiagen, Germany), and tested using a commercial Adenovirus PCR Kit (GeneProof, Brno, Czech Republic). The kit standards were used to generate the standard curves from the corresponding cycle threshold (Ct), and the unknown concentrations were calculated from the standard curve. The limit of detection was 2000 copies/ml.

#### Diagnosis of autoimmune hepatitis

Autoimmune hepatitis was diagnosed according to the protocol of Assiut University Hospital, as described previously [8, 17]. Briefly, IgG (total) human was done (Catalog # BMS2091, ThermoFischer Scientific). Detection of anti-smooth muscle antibodies (ASMA) was performed using ASMA ELISA Kit (MyBioSource, USA), and anti-nuclear antibody IgG using AccuDiag™ ELISA kit (Diagnostic Automation/Cortez Diagnostics INC. Woodland Hills, California, USA).

#### Diagnosis of metabolic diseases or other causes of ALF

Detailed medical histories excluded alcohol, drugs, or any hepatotoxins. Wilson’s disease was diagnosed by 24-h urinary copper and serum ceruloplasmin level. Doppler ultrasound and/or magnetic resonance imaging were used to diagnose acute Budd–Chiari syndrome.

Hepatitis was considered fulminant when patients developed impaired liver function, such as ascites, deep jaundice (total bilirubin > 10 mg/dL), prolonged prothrombin time > 5 s, albumin < 3 g/dL [18], and according to the guidelines of the European Association for the Study of the Liver (EASL) [19].

### Statistical analysis

The results of this study were assessed by the SPSS program (Chicago, Version 21). Categorical variables are expressed as frequencies and percentages. Quantitative results are presented as means ± standard deviations. Student’s *t*-test (parametric) and Mann–Whitney U test (non-parametric) were applied, and the chi-squared test was used for qualitative variables. P-value < 0.05 was considered significant. The correlation between different parameters was identified using Spearman’s correlation test.

## Results

### Demographic and clinical characteristics of the cohort

Forty-two (*n = *42) patients were included in this study, and they were divided into two groups according to the identified etiologies of acute hepatitis: Group 1, categorized as AHUE, included 24/42 patients (57.1%), while Group 2, the known hepatitis group, included 18/42 patients (42.9%). Cases of AHUE were defined by the exclusion of common viral hepatitis A–E, CMV, EBV, HSV1/2, and other causes, such as alcoholic hepatitis, drug-induced liver injury, autoimmune hepatitis, and Wilson’s disease (Table 1).

**Table 1:** The demographic and clinical characteristics of both groups

Item	Total (*n = *42) (*n* %)	Group 1 Acute hepatitis of unknown etiology (*n = *24) (*n* %)	Group 2 Acute hepatitis of identified etiology(*n = *18) (*n* %)	*P* value
Sex				0.044*
Males	26 (61.9%)	18 (75%)	8 (44.4%)	
Females	16 (38.1%)	6 (25%)	10 (55.6%)	
Age (mean ± SD)	34.55 ± 16.272	35.92 ± 17.177	32.72 ± 15.273	0.536
Range	14–72	16–72	14–64	
Clinical presentations				
Jaundice	39 (92.9%)	24 (100%)	15 (83.3%)	0.038*
Dark urine	26 (61.9%)	19 (79.2%)	7 (38.9%)	0.008*
Abdominal pain	22 (52.4%)	15 (62.5%)	7 (38.9%)	0.129
Fever	21(50%)	11 (45.8%)	10 (55.6%)	0.533
Vomiting	14 (33.3%)	5 (20.8%)	9 (50%)	0.047*
Diarrhea	5 (11.9%)	5 (20.8%)	0 (0%)	0.039*
Bleeding	3 (7.1%)	2 (8.3%)	1 (5.6%)	0.729
Encephalopathy	14 (33.3%)	7 (29.2%)	7 (38.9%)	0.508
Grade I	4 (9.5%)			
Grade II	1 (2.4%)			
Grade III	5 (11.9%)			
Grade IV	4 (9.5%)			
Ascites				
No	33 (78.6%)	19 (79.2%)	14 (77.8%)	0.632
Mild	8 (19%)	4 (16.7%)	4 (22.2%)	
Moderate	1 (2.4%)	1 (4.2%)	0 (0%)	
Course of hepatitis				
Acute hepatitis	20 (47.6%)	13 (54.2%)	8 (44.4%)	0.533
Fulminant	12 (28.6%)	7 (29.2%)	5 (27.8%)	
ACLF	10 (23.8%)	5 (20.8%)	5 (27.8%)	
Duration of symptoms (days): median (IQR)	14 (7.8)	13.5(8)	14(7)	0.331
Duration of hospitalization (days): mean ± SD	12.81 ± 8.037	12.63 ± 8.144	13.06 ± 8.120	0.866
Range	3–30	3–30	4–30	
Outcomes				
Alive	32 (76.2%)	18(75%)	14(77.8%)	0.834
Death	10 (23.8%)	6 (25%)	4 (22.2%)	

*ACLF* acute-on-chronic liver failure

Both represent Significant *p* value

* Significantly different values

The mean age of the total cohort was 34.55 ± 16.27 years and ranged from 14 to 72 years. The two groups did not differ significantly in terms of age, but they did in terms of gender ratio. The AHUE group contained 75% males, while Group 2 included 55.6% females (Table 1).

Jaundice and dark urine were the main clinical presentations in both groups (92.9% and 61.9%, respectively), followed by abdominal pain (52.4%). Fever was observed in 45.8% of the cases in Group 1 and in 55.6% of the cases in Group 2, and one-third of the cases developed hepatic encephalopathy. Overall, the clinical characteristics of both groups did not differ significantly, except for jaundice, dark urine, vomiting, and diarrhea, all of which were higher in the AHUE group. The groups did not differ in terms of acute and fulminant hepatitis or acute-on-chronic liver failure (ACLF). One case in each group underwent liver transplantation. The overall mortality rate was 23.8% (*n = *10) in both groups. Our cohort included four pediatric patients: two with acute HBV and two with AHUE, without reported mortality. The number of cases, particularly AHUE cases, doubled by 2022; however, it was not in the form of an outbreak or clustered cases.

### Laboratory parameters and ultrasound of the studied patients

The two groups did not significantly differ in terms of CBC, LFTs, and ultrasound (Table 2). However, LFTs such as serum total bilirubin, alanine transaminase, aspartate transaminase, and alkaline phosphatase levels were higher in the AHUE group compared with Group 2 (Table 2). Liver cirrhosis was detected in five cases in each group, and the incidences of hepatomegaly and splenomegaly were higher in the AHUE group (Table 2).

**Table 2:** Laboratory parameters and ultrasound of the studied patients

Item	Total (*n = *42)	Group 1 Acute hepatitis of unknown etiology (*n = *24) Mean ± SD	Group 2 Acute hepatitis of identified etiology (*n = *18) Mean ± SD	*P* value
WBCs (× 1000/mm^3^)	8.308 ± 5.3905	7.684 ± 4.1990	9.139 ± 6.7041	0.393
Hemoglobin (gm/dl)	12.515 ± 2.5410	12.683 ± 2.6563	12.300 ± 2.4442	0.638
Platelets (× 1000/mm^3^)	177.42 ± 82.911	175.56 ± 86.112	179.89 ± 80.842	0.869
Total bilirubin (umol/l) median (IQR)	221.3 (287)	231.2 (240)	163.5 (295)	0.127
Albumin (g/l)	30.564 ± 7.7973	30.583 ± 7.9941	30.539 ± 7.7568	0.986
ALT (IU/L) median (IQR)	658.5 (753.5)	785.5 (860)	419.5 (658.5)	0.409
AST (IU/L) median (IQR)	575.5 (494.2)	718 (498)	477 (572)	0.578
ALP (IU/L) median (IQR)	188.5 (139)	203.5 (181)	163 (78)	0.146
Prothrombin time (sec.)	20.033 ± 8.9170	20.296 ± 10.0706	19.683 ± 7.3708	0.829
INR	1.773 ± 0.8746	1.772 ± .9839	1.775 ± .731	0.992
Creatinine (umol/l) median (IQR)	70.7 (22.8)	70.7 (21.5)	63.5 (44.1)	0.930
Liver ultrasound				
Normal	23 (54.8%)	13 (54.2%)	10 (55.6%)	0.896
Fatty liver	1 (2.4%)	1 (4.2%)	0 (0%)	
Hepatomegaly	5 (11.9%)	3 (12.5%)	2 (11.1%)	
Hepatosplenomegaly	3 (7.1%)	2 (8.3%)	1 (5.6%)	
Cirrhosis	10 (23.8%)	5 (20.8%)	5 (27.8%)	

*WBCs* white blood cells, *ALT* alanine aminotransferase (normal: 0–45 IU/L); *AST* aspartate aminotransferase (normal: 0–34 IU/L); *ALP* alkaline phosphatase (normal: 46–116); *INR* international normalized ratio

### The etiology of acute hepatitis in the studied groups

We identified the causes of acute hepatitis in the studied groups. None of the cases developed respiratory symptoms or tested positive for COVID-19. Among the AHUE cases, five (20.8%) tested positive for adenovirus alone, and one case had a co-infection with HEV (Table 3). Other rare viral infections, such as HSV 1/2, coxsackie virus, and parvovirus B19, were not detected in any case. One case exhibited septicemia due to empyema, which was resolved by intercostal tube drainage and extensive broad-spectrum antibiotics. Meanwhile, in 18/24 (75%) of the patients, no specific pathogens were detected (Table 3).

**Table 3:** The identified causes of acute hepatitis in the studied groups

Unknown hepatitis (*n = *24) (*n* %)	Known hepatitis (*n = *18) (*n* %)
Adenovirus (5, 20.8%)	HBV (3, 16.7%)
Septicemia (1, 4.2%)	HCV(1, 5.6%)
Coxsackie virus (0%)	HEV (2, 11.1%)
Parvo virus B19 (0%)	EBV (1, 5.6%)
Idiopathic (18, 75%)	CMV (2, 11.1%)
	HSV 1/2 (0%)
	AIH (2, 11.1%)
	DILI (4, 22.2%)
	Co-infection
	HAV/HEV (2, 11.1%)
	HEV/adenovirus (1, 5.6%)

*HBV* hepatitis B virus, *HCV* hepatitis C virus, *HEV* hepatitis E virus, *EBV* Epstein Barr virus, *CMV* cytomegalo virus, *AIH* autoimmune hepatitis, *DILI* drug-induced liver injury, *HAV* hepatitis A virus

### Characteristics of adenovirus-infected patients

We determined the clinical and demographic features of the patients infected with adenovirus (Table 4). They included four males and two females, with an age range of 21–72 years. Fever and abdominal pain were observed in two patients, jaundice in four, hepatic encephalopathy in one, hypoalbuminemia in four, and coagulopathy in four. One case was diagnosed as acute hepatitis, two as fulminant hepatitis, and the remaining three as ACLF. Only one of these patients died from decompensated liver cirrhosis. These cases were not linked epidemiologically (Table 4).

**Table 4:** Characteristics of patients infected by adenovirus in the study

Items	Case 1	Case 2 (HEV/adenovirus)	Case 3	Case 4	Case 5	Case 6
Age	72	21	35	34	33	45
Sex	Male	Female	Female	Male	Male	Male
Duration of symptoms (days)	7	14	10	3	30	5
Clinical presentation	Abdominal pain and dark urine	Fever and jaundice	Jaundice and dark urine	Jaundice and dark urine	Fever, abdominal pain and dark urine	Jaundice and hepatic enchephalopathy grade 4
Viral Load copies/ml	2.79 × 10^4^	1.21 × 10^5^	2.13 × 10^8^	2.096 × 10^3^	6.23 × 10^7^	7.27 × 10^7^
Total bilirubin (umol/l)	88	180	244	33.4	149	591.00
ALT(IU/L)	810	362	659	185	1763	81.0
AST (IU/L)	153	445	709	86	1210	362
Albumin (g/L)	32	20	23	37	20	21
Prothrombin time (sec.)	14.9	18	21.4	12.4	32.4	29.8
INR	1.27	1.5	1.79	1.03	2.6	2.5
Ultrasound	Cirrhosis	Hepatosplenomegaly, mild ascites	Normal	Normal	Cirrhosis, mild ascites	Cirrhosis and moderate ascites
Course of hepatitis	ACLF	Fulminant	Fulminant	Acute hepatitis	ACLF	ACLF
Outcome	Improved	Improved	Improved	Improved	Improved	Died

### Correlation of clinical and laboratory parameters with the outcomes of acute hepatitis

We assessed the factors affecting the outcomes of acute hepatitis. We found that they were significantly positively correlated with the grade of hepatic encephalopathy, total bilirubin level, and INR, while significantly negatively correlated with albumin level (Table 5). Meanwhile, the etiology of acute hepatitis and the duration of symptoms or hospitalization did not significantly correlate with the outcome (Table 5).

**Table 5:** Correlation between clinical and laboratory parameters with the outcomes of acute hepatitis

Variable		Outcome
Age	*r* value	0.164
	*P* value	0.300
Encephalopathy grade	*r* value	0.683
	*P* value	0.000**
Duration of symptoms	*r* value	0.03
	*P* value	0.850
Duration of hospitalization	*r* value	− 0.110
	*P* value	0.488
Etiology of hepatitis	*r* value	0.166
	*P* value	0.294
WBCs	*r* value	0.224
	*P* value	0.154
Hemoglobin	*r* value	0.053
	*P* value	0.743
Platelets	*r* value	− 0.145
	*P* value	0.359
Total bilirubin	*r* value	0.581
	*P* value	0.000**
ALT	*r* value	− 0.231
	*P* value	0.142
AST	*r* value	− 0.023
	*P* value	0.885
Albumin	*r* value	− 0.346
	*P* value	0.025*
ALP	*r* value	0.133
	*P* value	0.427
INR	*r* value	0.489
	*P* value	0.001**
Creatinine	*r* value	0.300
	*P* value	0.054

*WBCs* white blood cells, *ALT* alanine aminotransferase; *AST* aspartate aminotransferase; *ALP* alkaline phosphatase, *INR* international normalized ratio

**P value: significant *p* value

## Discussion

Acute non-A–E, seronegative, or indeterminate hepatitis differs from AHUE. The former can be caused by viruses such as CMV, EBV, HSV1/2, drug-induced liver injury, autoimmunity, and Wilson’s disease [4]. AHUE, on the other hand, excludes all of these causes. ALF, caused by non-A–E viral hepatitis, represents about one-third of acute hepatitis cases in India [2]. Indeterminate hepatitis is known to be more common in pediatric patients than in adults [3].

Recently, the World Health Organization reported cases of severe AHUE in children from Scotland, the UK, and other countries. Importantly, the detection of adenovirus and SARS-CoV-2 in these children confirmed new emerging causes for acute hepatitis [12]. In a recent study conducted in Egyptian hospitals, HEV was shown to be responsible for 10% of AHUE cases [8]. Prior to this, HEV had not been diagnosed in Egypt; therefore, the authors recommended its inclusion in the AHUE screening protocol. However, 90% of AHUE cases in Egypt remain uncharacterized. Herein, we aimed to characterize them and identify new etiological agents, thereby, providing the first report describing the etiology and characteristics of AHUE patients in Egypt.

In the current study, 24/42 (57.1%) cases were labeled as AHUE and 75% of them still lack a definitive etiology, which indicates a higher prevalence than the previous analysis, which reported 20% ALF cases with cryptogenic etiology [3]. The previous findings indicate that unidentified agents/pathogens that could cause AHUE still exist in Egypt, and further studies are required to identify them. In this study, AHUE patients were predominantly male (75%), and the mean age was about 35 years, which is comparable to the age reported by two other studies: one on indeterminate hepatitis (40 years) and the other on non-A–E-ALF patients (28.8 ± 12.0 years). However, female cases were predominant in these two reports [20, 21].

In the current study, jaundice (100%), dark urine (79.2%), and abdominal pain (62.5%) were frequently reported in AHUE patients, while fever was less frequent compared with cases of known hepatitis. Our findings concur with reports on the current outbreaks, wherein gastrointestinal symptoms were common, including jaundice, vomiting, pale stools, and diarrhea, while fever was reported less often [13, 22].

ALF developed in about half of our cohort, and AHUE patients showed a mortality rate similar to that of patients with known hepatitis (25% vs. 22%, respectively). The overall mortality was lower than 30% which was the mortality rate of indeterminate hepatitis reported by the Acute Liver Failure Study Group (ALFSG) [21]. This variation in the mortality rate could be attributed to the small sample size in the present study.

We characterized the etiology of the known hepatitis cases. Besides known viral hepatitis, EBV infection was detected in one case and CMV infection in two cases. EBV and CMV cause acute hepatitis in immunocompetent patients, which could either be asymptomatic with elevated transaminases, icteric hepatitis with a self-limited course, or ALF [23]. EBV can induce hepatitis in about 75% of patients, with a two- to three-fold increase in alanine transaminase levels, which return to normal after about 3 weeks [24]. Rarely, patients could have 5–tenfold elevated transaminase levels, with jaundice reported in less than 5% of them [25]. Hepatic injury is usually transient and self-limiting, but cases of fatal liver failure in immunocompetent patients have been reported [26, 27]. CMV infection causes liver abnormalities in 30–80% of patients [28, 29], and these may present with ALF requiring liver transplantation [30].

In this study, adenovirus was detected in 20.8% of AHUE cases (*n = *5) and co-infection with HEV was discovered in one case (5.6%). In recent cases from the UK, adenovirus was reported in 72% of them and co-infection with SARS-CoV-2 in 15% [31]. None of our cases displayed clinical symptoms of or tested positive for COVID-19. Co-infection of adenovirus and HEV could be attributed to the common route of transmission of both viruses (fecal–oral). Co-infections associated with hepatitis have been previously reported, e.g. coxsackie virus and EBV [32]. Adenovirus infections are easily transmissible, and the fate of infection could vary from a self-limiting course to a severe lethal course and also outbreaks [33, 34]. Half of the adenovirus-infected cases in our study had developed liver cirrhosis and liver failure was reported in 5/6 (83.3%) cases. The immune dysfunction which develops during liver cirrhosis could explain the high frequency of adenovirus infection in these cases. Other studies reported the association of adenovirus infection and development of fulminant hepatitis [35, 36]. In this study, we used a universal primer and probe that target a conserved region in different adenovirus serotypes [37]. The striking point of our study is the emergence of adenovirus infection among adults. These infections are common among children, but not adults. Further studies are needed to characterize the adenovirus strains circulating in Egypt and to identify the causes underlying the emergence of this pathogen in adults.

Investigations for rare viral hepatitis, such as those caused by HSV 1/2, parvovirus B19, and coxsackie virus, were negative. HSV, which represents < 1% of all causes of ALF and < 2% of all viral causes of ALF, induces massive hepatocellular necrosis. It commonly appears in immunosuppressed patients or during pregnancy; however, it has been reported in 25% of the immunocompetent patients in a study [38]. Parvovirus B19, less common in adult patients, is recognized to cause self-limiting acute hepatitis with an indolent course and can infect immunocompetent individuals. Acute B19 infection is diagnosed by the consistent detection of anti-parvovirus IgM and IgG [3]. It was reported in a small case series associated with aplastic anemia. The exact mechanism of hepatotoxicity is not well-understood, however, caspase 3-mediated apoptosis is a proposed theory [39]. Coxsackie virus, a member of the genus Enterovirus, is a rare cause of hepatitis, but coxsackie virus type B and A9 have been reported to induce hepatitis [40].

Our study had some limitations, such as a small cohort size. However, patients with known acute hepatitis are usually not admitted to the hospital except when it is severe, or the symptoms deteriorate. Thus, cases of known hepatitis in this study represent hospitalized cases rather than the actual community-based prevalence of the different etiologies. Studies on AHUE in Egyptian children are still lacking. Therefore, the actual prevalence of AHUE in Egyptian children is not known. Furthermore, further studies should compare the results of AHUE in children and adults in Egypt. In addition, we could not study the genome sequence of the identified adenovirus. Since none of the included patients showed symptoms of COVID-19, future studies need to assess the risk of SARS-CoV-2 and adenovirus co-infection to the Egyptian population. Importantly, we analyzed the samples for known causes of acute hepatitis using traditional serological and molecular approaches. Further studies should employ higher sensitivity approaches, such as next-generation sequencing, to identify other potential causes of acute hepatitis.

Identifying the etiologies of acute hepatitis could be challenging. Liver biopsies may be helpful, but are not always available, and could be diagnostic in only a few cases like HSV-based hepatitis. Further investigations on novel rare causes of AHUE will be needed, focusing on viral hemorrhagic fevers, parainfluenza virus, sapovirus, norovirus, bocavirus, etc. Early diagnosis of decompensation or fulminant hepatitis is necessary for an early recommendation of liver transplantation [41]. The current study confirmed this, as the outcome of acute hepatitis was correlated with laboratory parameters related to severe impairment of liver function, while the etiology of acute hepatitis was not.

## Conclusions

In this first report of sporadic AHUE cases in Egypt, adenovirus was detected in adults. Further multi-center studies are required to determine the prevalence of this newly emerging viral hepatitis. The diagnosis of adenovirus in this study serves as an alarm to consider this diagnosis while treating AHUE, so that further emergence of adenovirus outbreaks, like the recently reported cases across Europe, can be avoided.

## Data Availability

All the data are present in the main text. For further inquiries, please contact the first and corresponding authors.

## References

[1] Bernal W (2013). Lessons from look-back in acute liver failure? A single centre experience of 3300 patients. J Hepatol.

[2] Shalimar, Achraya SK, Lee WM (2013). Worldwide differences in acute liver failure. Future Med.

[3] Brennan P, Donnelly MC, Simpson KJ (2018). Systematic review: non A-E, seronegative or indeterminate hepatitis; what is this deadly disease?. Aliment Pharmacol Ther.

[4] Ganger DR (2018). Acute liver failure of indeterminate etiology: a comprehensive systematic approach by an expert committee to establish causality. J Am Coll Gastroenterol.

[5] Nakao M (2018). Nationwide survey for acute liver failure and late-onset hepatic failure in Japan. J Gastroenterol.

[6] Donnelly M (2017). Acute liver failure in Scotland: changes in aetiology and outcomes over time (the Scottish Look-Back Study). Aliment Pharmacol Ther.

[7] Mendizabal M (2014). Changing etiologies and outcomes of acute liver failure: perspectives from 6 transplant centers in Argentina. Liver Transpl.

[8] Sayed IM (2021). Clinical outcomes and prevalence of hepatitis E Virus (HEV) among non-A-C hepatitis patients in Egypt. Infect Drug Resist.

[9] El-Mokhtar MA (2021). Evaluation of hepatitis E antigen kinetics and its diagnostic utility for prediction of the outcomes of hepatitis E virus genotype 1 infection. Virulence.

[10] Kumar R (2010). Antituberculosis therapy–induced acute liver failure: magnitude, profile, prognosis, and predictors of outcome. Hepatology.

[11] Schiødt FV (2003). Viral hepatitis-related acute liver failure. Am J Gastroenterol.

[12] https://www.who.int/emergencies/disease-outbreak-news/item/acute-hepatitis-of-unknown-aetiology--the-united-kingdom-of-great-britain-and-northern-ireland. Accessed 13 Sept 2022.

[13] WHO. Multi-country acute, severe hepatitis of unknown origin in children. Disease Outbreak News. 2022. https://www.who.int/emergencies/disease-outbreak-news/item/2022-DON400. Accessed 13 Sept 2022.

[14] Gutierrez Sanchez LH, et al. A case series of children with acute hepatitis and human adenovirus infection. New Engl J Med. 2022. 10.1056/NEJMoa2206294 PMC9808750 35830653

[15] Kelgeri C (2022). Clinical spectrum of children with acute hepatitis of unknown cause. New Engl J Med.

[16] Barrett-Muir WY (1998). Evaluation of the murex hybrid capture cytomegalovirus DNA assay versus plasma PCR and shell vial assay for diagnosis of human cytomegalovirus viremia in immunocompromised patients. J Clin Microbiol.

[17] El-Mokhtar MA (2021). The unmet needs of hepatitis E virus diagnosis in suspected drug-induced liver injury in limited resource setting. Front Microbiol.

[18] Tassopoulos NC (2008). Clinicopathological features and natural history of acute sporadic non-(A-E) hepatitis. J Gastroenterol Hepatol.

[19] Wendon J (2017). EASL Clinical Practical Guidelines on the management of acute (fulminant) liver failure. J Hepatol.

[20] Acharya SK (2020). Acute liver failure of non–AE viral hepatitis etiology—profile, prognosis, and predictors of outcome. J Clin Exp Hepatol.

[21] Ganger DR, et al. Acute liver failure of indeterminate etiology: a comprehensive systematic approach by an expert committee to establish causality. Am J Gastroenterol. 2018;113:1319. 10.1038/s41395-018-0160-2 PMC9252260 29946176

[22] Baker JM (2022). Acute hepatitis and adenovirus infection among children—Alabama, October 2021–February 2022. Morb Mortal Wkly Rep.

[23] Bunchorntavakul C, Reddy KR (2020). Epstein-Barr virus and cytomegalovirus infections of the liver. Gastroenterol Clin.

[24] Kofteridis DP (2011). Epstein Barr virus hepatitis. Eur J Intern Med.

[25] Crum NF (2006). Epstein Barr virus hepatitis: case series and review. South Med J.

[26] Ader F (2006). Fulminant Epstein-Barr virus (EBV) hepatitis in a young immunocompetent subject. Med Mal Infect.

[27] Feranchak AP (1998). Fulminant Epstein-Barr viral hepatitis: orthotopic liver transplantation and review of the literature. Liver Transpl Surg.

[28] Bonnet F, et al. Clinical and laboratory findings of cytomegalovirus infection in 115 hospitalized non-immunocompromised adults. Ann Med Intern. 2001. 11474369

[29] Wreghitt T (2003). Cytomegalovirus infection in immunocompetent patients. Clin Infect Dis.

[30] Yu Y-D (2013). Cytomegalovirus infection-associated fulminant hepatitis in an immunocompetent adult requiring emergency living-donor liver transplantation: report of a case. Surg Today.

[31] Cevik M (2022). Acute hepatitis of unknown origin in children. BMJ.

[32] Atti V, Anderson NM, Day MB (2017). Coxsackie myocarditis and hepatitis with reactivated epstein-bar virus (EBV): a case report. Am J Case Rep.

[33] Alharbi S (2012). Epidemiology of severe pediatric adenovirus lower respiratory tract infections in Manitoba, Canada, 1991–2005. BMC Infect Dis.

[34] Lewis PF (2009). A community-based outbreak of severe respiratory illness caused by human adenovirus serotype 14. J Infect Dis.

[35] Wang WH, Wang HL (2003). Fulminant adenovirus hepatitis following bone marrow transplantation: a case report and brief review of the literature. Arch Pathol Lab Med.

[36] Ronan B (2014). Fulminant hepatitis due to human adenovirus. Infection.

[37] Heim A (2003). Rapid and quantitative detection of human adenovirus DNA by real-time PCR. J Med Virol.

[38] Norvell JP (2007). Herpes simplex virus hepatitis: an analysis of the published literature and institutional cases. Liver Transpl.

[39] Poole BD (2006). Apoptosis of liver-derived cells induced by parvovirus B19 nonstructural protein. J Virol.

[40] Moreau B (2011). Hepatitis and Encephalitis due to Coxsackie Virus A9 in an Adult. Case Rep Gastroenterol.

[41] Bernal W, Wendon J (2013). Acute liver failure. N Engl J Med.

